# Human brain metastatic stroma attracts breast cancer cells via chemokines CXCL16 and CXCL12

**DOI:** 10.1038/s41523-017-0008-8

**Published:** 2017-03-02

**Authors:** Brile Chung, Ali A. Esmaeili, Sailesh Gopalakrishna-Pillai, John P. Murad, Emily S. Andersen, Naveen Kumar Reddy, Gayathri Srinivasan, Brian Armstrong, Caleb Chu, Young Kim, Tommy Tong, James Waisman, John H. Yim, Behnam Badie, Peter P. Lee

**Affiliations:** 1 Department of Immuno-Oncology, City of Hope, Duarte, CA USA; 2 Light Microscopy Imaging Core, City of Hope, Duarte, CA USA; 3 Department of Pathology, City of Hope, Duarte, CA USA; 4 Department of Medical Oncology & Therapeutics Research, City of Hope, Duarte, CA USA; 5 Department of Surgery, City of Hope, Duarte, CA USA

## Abstract

The tumor microenvironment is composed of heterogeneous populations of cells, including cancer, immune, and stromal cells. Progression of tumor growth and initiation of metastasis is critically dependent on the reciprocal interactions between cancer cells and stroma. Through RNA-Seq and protein analyses, we found that cancer-associated fibroblasts derived from human breast cancer brain metastasis express significantly higher levels of chemokines CXCL12 and CXCL16 than fibroblasts from primary breast tumors or normal breast. To further understand the interplay between cancer cells and cancer-associated fibroblasts from each site, we developed three-dimensional organoids composed of patient-derived primary or brain metastasis cancer cells with matching cancer-associated fibroblasts. Three-dimensional CAF aggregates generated from brain metastasis promote migration of cancer cells more effectively than cancer-associated fibroblast aggregates derived from primary tumor or normal breast stromal cells. Treatment with a CXCR4 antagonist and/or CXCL16 neutralizing antibody, alone or in combination, significantly inhibited migration of cancer cells to brain metastatic cancer-associated fibroblast aggregates. These results demonstrate that human brain metastasis cancer-associated fibroblasts potently attract breast cancer cells via chemokines CXCL12 and CXCL16, and blocking CXCR6-CXCL16/CXCR4-CXCL12 receptor–ligand interactions may be an effective therapy for preventing breast cancer brain metastasis.

## Metastasis: brain metastases fuel further spread

Breast cancer metastases to the brain secrete signaling molecules that promote additional cancer cells to migrate there. Peter P. Lee and colleagues from the City of Hope in Duarte, California, USA, analyzed protein and gene expression levels in brain metastases, and showed that it is the stromal cells (support cells such as fibroblasts), rather than the cancer cells themselves, that are the source of these homing signals. When compared against stromal cells derived from primary breast tumors or healthy breast tissue, they found that the stromal cells that had lodged themselves in the brain expressed the highest levels of CXCL12 and CXCL16, two chemokines involved in cell movement. Using three-dimensional aggregates, the researchers showed that these metastatic stromal cells promoted cancer cells migration more potently than stromal cells from primary tumors or normal breast tissues. Blocking the chemokine activity or that of its receptor impaired cancer cell movement, suggesting a possible therapeutic strategy for preventing brain metastasis in patients with breast cancer. These results highlight the importance of the tumor microenvironment and stromal cells in the metastasis process of breast cancer.

## Introduction

Brain metastasis is the most lethal outcome of breast cancer, leading to death within 4–6 months in 10–15% of patients once detected.^1, 2^ For brain metastasis to occur, cancer cells from the primary tumor must migrate to the brain, traverse the blood–brain barrier, and proliferate within the brain parenchyma.^3^ Emerging data suggest that outcome of metastasis is influenced by the specific organ microenvironment stromal cells that permit the effective colonization and growth of circulating tumor cells.^4^ We hypothesized that mesenchyme-derived fibroblasts, the major cell population of tumor stroma, promote invasion, survival, and proliferation of migrating cancer cells to facilitate breast cancer brain metastasis.

Conventional methods to model the metastatic process ex vivo mainly involve two-dimensional (2D) monolayer in vitro systems, which do not recapitulate the three-dimensional (3D) in vivo microenvironment. Cell–cell and cell–extracellular matrix (ECM) interactions in 3D spatial environment are critical for understanding the complex cross-talk mechanisms between cancer and stromal cells. For example, both gene and protein expressions in an ex vivo 3D culture system appear to conserve various paracrine-dependent cellular interactions that occur in vivo microenvironment.^5–7^ Furthermore, studies have shown that testing of chemotherapy treatments or immunotherapies based on 2D monolayer systems does not correspond with results in an in vivo setting, further demonstrating the limitations of 2D monolayer systems.^8^ Hence, developing and testing the effectiveness of novel therapies for breast cancer in vitro require recreation of the 3D breast cancer microenvironment composed of stroma and cancer cells, ideally derived from the same patient, as one functional unit.

Cancer-associated fibroblasts (CAFs) have been shown to produce various chemokines to facilitate angiogenesis and cancer cell migration.^9^ To investigate the role of CAFs in breast cancer brain metastasis, we isolated and expanded fibroblasts derived from normal breast, primary, and brain metastatic tumor tissues. Utilizing 3-D ex-vivo aggregates composed of different CAFs with cancer cells, we evaluated the expression of various chemokines and growth factors by RNA-Seq, real-time quantitative qPCR, immuno-histochemical staining, and enzyme-linked immunosorbent assay (ELISA). These studies showed that metastatic CAFs from brain metastases produce high levels of chemokines CXCL12 and CXCL16, promoting the migration of patient-specific breast cancer cells in a 3-D aggregate system. Moreover, blocking of CXCR4, the chemokine receptor for CXCL12, and neutralization of CXCL16, the ligand for CXCR6 in patient-specific cancer cells significantly prevented the migration of cancer cells to the tumor microenvironment (TME). These novel findings from our 3D CAF aggregate system provide proof of principle that chemokine modulation represents an effective therapeutic strategy to prevent tumor progression and metastasis.

## Results

### Isolation of breast cancer cells and CAFs from patient tumor tissues

**Fig. 1:**
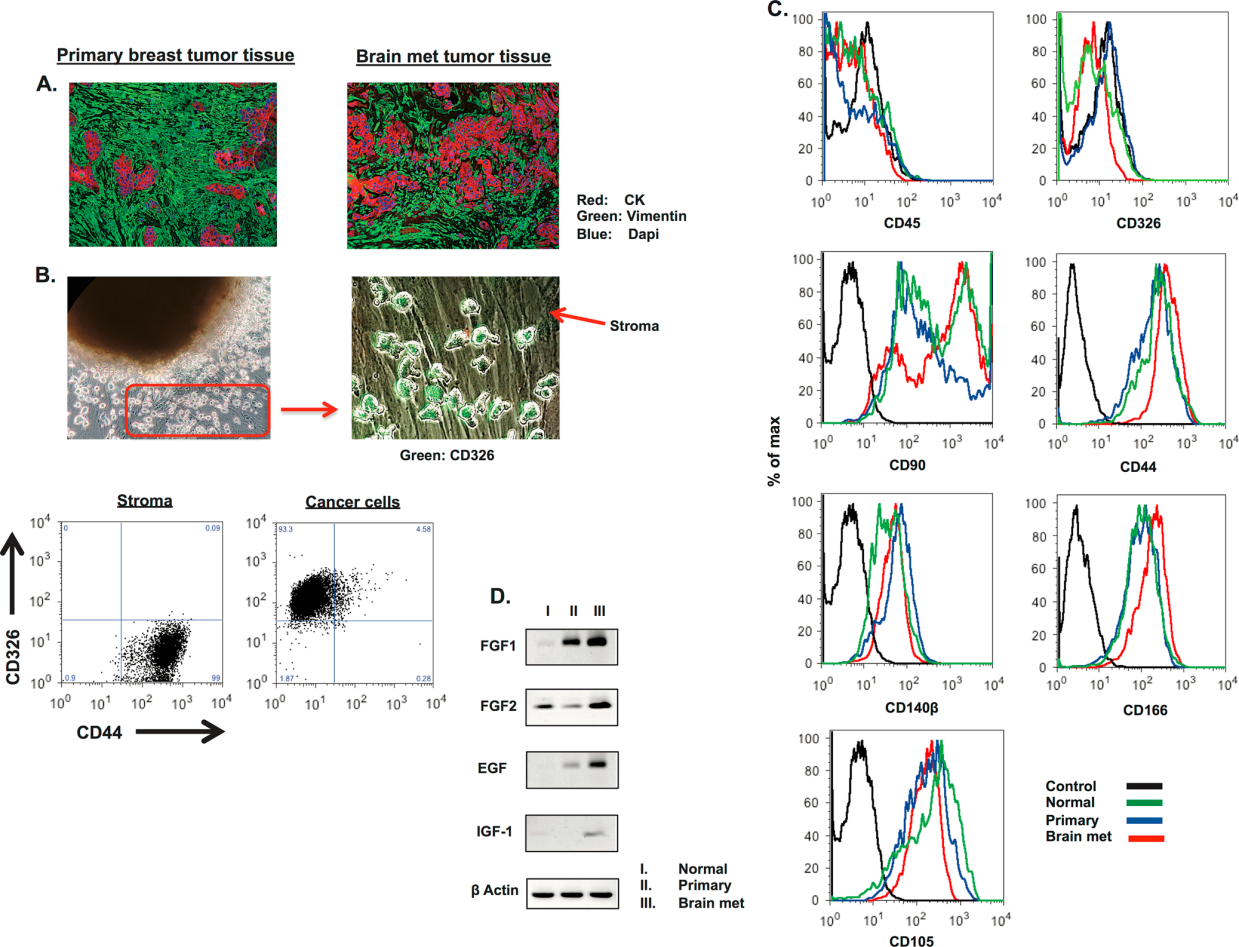
Characterization of fibroblasts isolated from primary and brain met breast tumor in culture. **a** Immunohistochemistry was performed to determine the prevalence of CAFs (vimentin+) surrounding breast cancer cells (CK+). **b** Morphology of CAFs (CAS) and breast cancer cells growing in tissue culture, 2 weeks after plating human breast tumor fragments (*green color* represents CD326+ cancer cells and a *red arrow* indicates CD44+ CD326- fibroblasts. **c** At 2–4 weeks, normal human breast fibroblast, primary CAS, and brain met CAS surface marker expression was analyzed by FACS. **d** Gel electrophoresis RT PCR data demonstrates relative growth factor expression of FGF-1, FGF-2, EGF, and IGF-1 in normal, primary and metastatic aggregate stroma

To study cancer cells and CAFs derived from breast tumors, we obtained fresh human breast tumor tissues from six primary and six metastatic patients following surgery or biopsy (Table 1). As controls, we also obtained six normal breast tissue samples from either the contralateral breast of breast cancer patients, or patients who underwent prophylactic mastectomy. Histological analysis of both human primary breast and brain metastatic tumor samples showed the presence of vimentin-positive stromal cells surrounding cytokeratin-positive breast cancer cells (Fig. 1a). To study these cells and develop an ex-vivo culture system that allows expansion of both patient-specific breast cancer cells and CAFs, human breast tumor tissue was mechanically dissociated into small fragments, and plated onto tissue culture plate in medium supplemented with epidermal and keratinocyte growth factor. Within 2 weeks, both CD326+ CD44− cancer cells and CD326− CD44+ CAFs expanded by outgrowth from the initial tumor fragments (Fig. 1b). To investigate whether CD326− CD44+ adherent fibroblasts express mesenchyme-derived surface markers, we performed immunophenotypic characterization of the monolayer generated in breast tumor fragment cultures after 3 weeks by flow cytometry. Nearly all the ex vivo expanded mesoderm-derived fibroblasts from normal breast, and CAFs from primary and brain metastatic tumors expressed the common mesenchyme markers CD44, CD90, CD105, CD166, and CD140β (Fig. 1c). In contrast, CD326+ breast cancer cells did not display the surface markers expressed by CAFs (Supplemental Fig. 1).

**Table 1:** Patient characteristics

Number of patients	Median age of patients	Mean age of patients				
5	32	37				
Sample	Type	Molecular subtype (ER, PR, Her2)	Tumor grade	Cancer stage	Age	BRCA
BC56	Normal				48	(+)
BC78	Normal				32	(+)
BC82	Normal				32	
BC97	Normal				47	
BC 131	Normal				25	(+)

Number of patients	Median age of patients	Mean age of patients				
8	58	55				
Sample	Type	Molecular subtype (ER, PR, Her2)	Tumor grade	Cancer stage	Age	
BC68	Primary	(+), (+), (−)	II	IA	64	
BC80	Primary	(+), (+), (−)	III	IIA	29	
BC84	Primary	(+), (−), (−)	III		72	
BC 95	Primary	(+), (−), (+)	III	IA	50	
BC105	Primary	(+), (+), (−)	III	IIA	71	
BC108	Primary	(+), (+), (−)	II	IIA	39	
BC153	Primary	(+), (+), (−)	I	IIIA	59	
BC155	Primary	(+), (+), (−)	II	IIB	57	

Number of patients	Median age of patients	Mean age of patients				
7	59	59				
Sample	Type	Molecular subtype (ER, PR, Her2)	Tumor grade	Cancer stage	Age	
BC25	Brain Met	(−), (−), (+)			66	
BC55	Brain Met	(−), (−), (+)			54	
BC66	Brain Met	(+), (+), (+)			52	
BC70	Brain Met	(+), (+), (−)			63	
BC 122	Brain Met	(+), (+), (+)			59	
BC137	Brain Met	(+), (+), (−)			54	
BC 156	Brain Met	(−), (−), (+)			62	

Primary breast tumor patients are categorized based on molecular subtypes, tumor grade, cancer stage, and age. Patients with brain metastasis are categorized based on molecular subtypes and age.

Both semi-quantitative and quantitative PCR analysis demonstrated that Epidermal Growth Factor (EGF), Fibroblast Growth Factor (FGF), and Insulin-like Growth Factor (IGF-1) (factors known to support growth of cancer cells) were expressed by both primary tumor and brain metastasis CAFs (Fig. 1d and Supplemental Fig. 2). This provides evidence that cultured CAFs produce factors important for maintenance of patient-specific breast cancer cells. Since bone marrow-derived mesenchymal stem cells (MSCs) are known to reside within breast TME and express similar surface markers as CAFs, trans-differentiation assays were performed to determine if some of the CAF populations were capable of undergoing adipogenesis as observed in MSCs. In addition, we further investigated the expression of STRO-1, the surface antigen known to express by bone marrow MSCs. Our data showed that CAFs derived from primary breast tumor and brain metastasis express higher levels of STRO-1, and can differentiate into adipocytes, suggesting our CAF culture contains MSC-like cell populations (Supplemental Fig. 3).^10, 11^

### Generation of human breast tumor-derived CAF aggregates

**Fig. 2:**
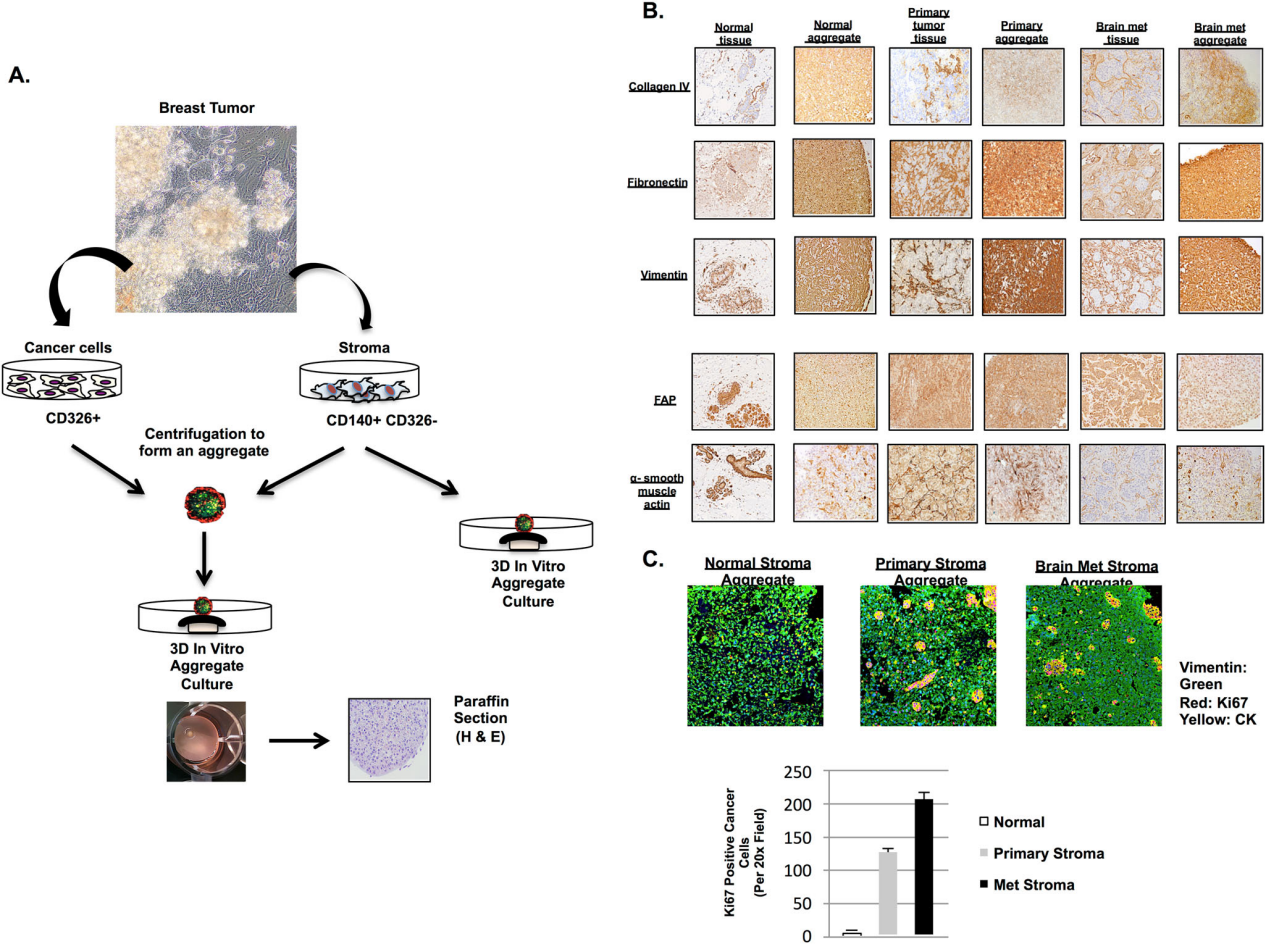
Generation of 3-D human breast cancer microenvironment in vitro. **a** Schematic representation for generation of 3-D human breast stroma aggregates for in vitro model. Human breast cancer associated breast stromal cells were generated from patient specific primary or brain met tumor tissues ex vivo. Aggregates were then cultured on nucleopore membranes floating in D10 medium supplemented with human epidermal growth factor for 2 weeks for in vitro analyses. **b** Immuno-histochemical staining comparison between human breast cancer tissues and cancer-associated stromal aggregates. Paraffin-embedded stromal aggregates were sectioned and stained for vimentin, and activated fibroblast markers including alpha smooth muscle and fibroblast activating protein (FAP). Expression of ECM components was analyzed in both tissue section and aggregates. Antibody staining directed against fibronectin and collagen IV showed the presence of ECM in all aggregates. **c** Immuno-fluorescent antibody staining against Ki67 (*red*), cytokeratin (*yellow*) and vimentin (*green*) in patient-derived aggregates composed of cancer cells mixed with either normal, primary or metastatic stroma. *Bar graph* illustrates relative expression of Ki67 in cancer cells combined with either normal, primary or metastatic stroma patient samples

2-D culture models do not fully replicate complexities in tumor tissues, such as multi-dimensional cellular structure, extracellular matrixes, and divergent gene expression patterns.^12^ Hence, we generated 3-D aggregates from cells cultured out of normal breast tissue, primary and metastatic tumors to recapitulate complexities displayed by the human TME. Normal breast fibroblast and patient-specific CAF aggregates were created by centrifugation of monolayers generated from tissue culture, followed by further culturing on nucleo-pore filters (Fig. 2a).

In order to demonstrate the ability of 3-D CAF aggregates to produce, and maintain ECM and CAF markers, cell aggregates were cultured and paraffin-sectioned for histological analyses. H&E stains of normal breast and tumor samples showed morphological similarities with their corresponding 3-D CAF aggregates (Supplemental Fig. 4). IHC stains showed that important ECM components, such as Collagen IV and Fibronectin, were preserved in all aggregates as compared to fresh human breast tumor tissues (Fig. 2b). Expression of fibroblastic activating protein (FAP) and alpha smooth muscle actin (*α*-SMA) has been described in myofibroblasts and CAF.^13–15^ As expected, FAP and *α*-SMA expressing cells were more prevalent in primary and brain metastasis CAF aggregates when compared to normal fibroblast aggregates (Fig. 2b). To further investigate whether FAP+ *α*-SMA+ cells detected from brain metastasis aggregates originated from cell types of the central nervous system (CNS), such as astrocytes and ependymal cells, we examined the expression of glilal fibrillary acidic protein (GFAP) in brain metastasis aggregates (Supplemental Fig. 5). CAFs from brain metastasis do not express GFAP, suggesting that they are of non-CNS origin.^16^

To demonstrate whether our CAF aggregate system can maintain and promote proliferation of cancer cells, we generated CAF aggregates mixed with breast cancer cells and measured Ki-67 expression in cancer cells. Here, we generated patient-derived cancer cells with patient-derived CAF aggregates in order to more fully mimic the natural TME setting. Data shown in Fig. 2c demonstrate that our 3D co-culture system supports proliferation of patient-derived cancer cells. We detected significantly higher numbers of Ki-67-positive cancer cells in primary and metastatic CAF aggregates than from normal breast fibroblasts aggregates (Fig. 2c). Mesenchyme-derived growth factors known to promote cancer cell proliferation (EGF, FGF1, FGF2, and IGF-1) were expressed in different CAF populations to different levels (Fig. 1d and Supplemental Fig. 2). Overall, these results showed that the ex vivo CAF 3-D aggregates system served as a sufficient ECM producing microenvironment and provided growth factors capable of cancer cells proliferation.

### mRNA level expression and histological analysis of chemokines in primary tumor and brain metastasis-derived human breast CAF 3-D aggregates

**Fig. 3:**
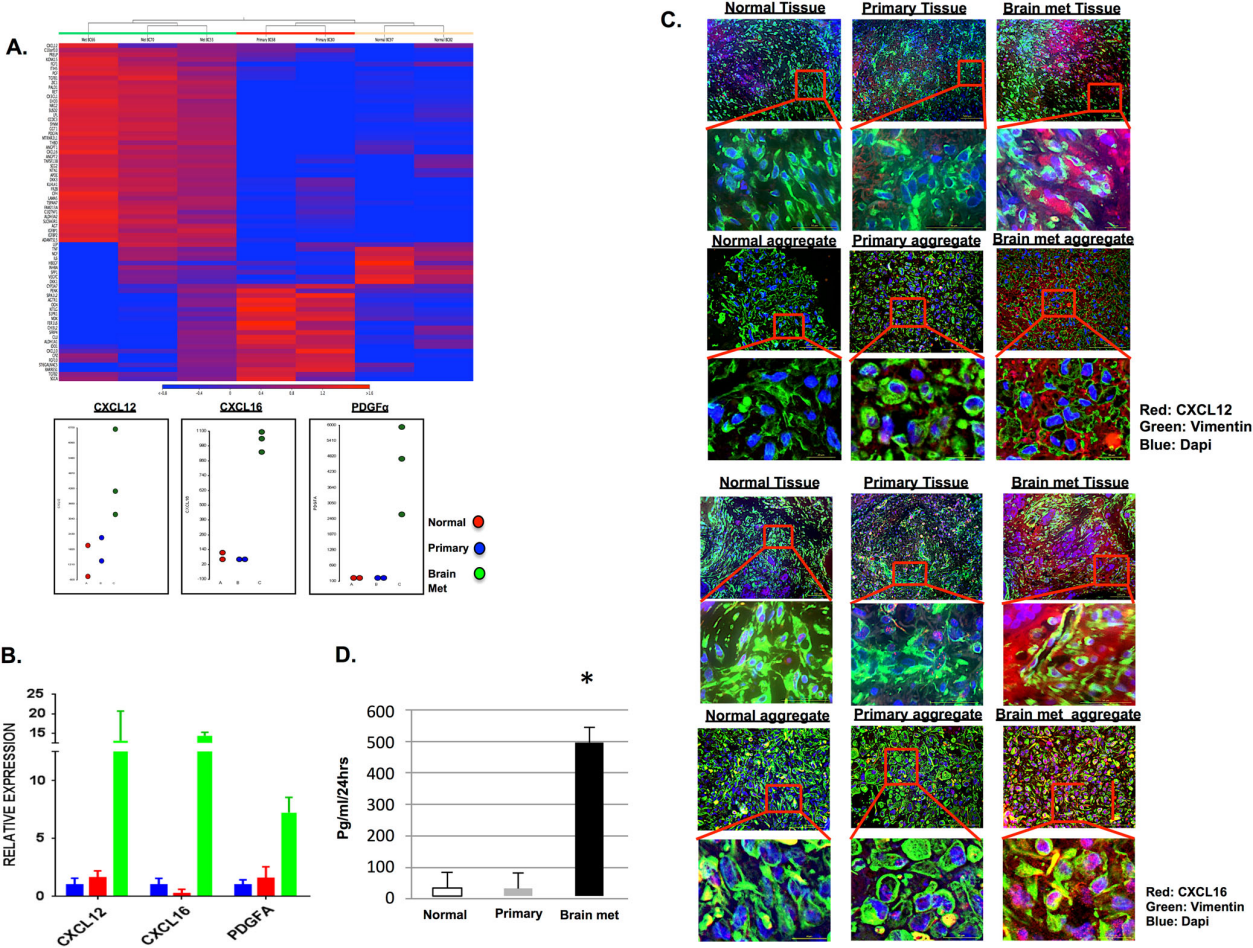
Gene and protein expression analyses of 3-D human breast cancer CAF aggregates. **a** Heat map of hierarchical cluster analysis of RNA seq data derived from the normal, primary breast tumor CAF, and brain met CAF aggregates. BC82 and BC97 were used for the normal aggregate group; BC68 and BC80 were used for the primary tumor aggregate group; BC66, BC70 and BC55 were used for the brain metastasis aggregate group (*see* Table 1). Individual gene *dot plots* showing changes in mean expression levels of transcripts PDGFα, CXCL12, and CXCL16. **b** Quantitative RT-PCR validation of relative changes in expression levels of the CXCL12, CXCL16, and PDGFα. **c** Immuno-fluorescent staining directed against CXCL12 and CXCL16 expression from patient tissue and patient-derived CAF aggregate. Representative immuno-fluorescence images show Vimentin (*green*) and CXCL12/CXCL16 (*red*) expression in both patient tissues and patient-derived stromal aggregates. *Scale bar* for zoomed images represent 50 µm. **d** Measurement of soluble CXCL16 in media of patient-derived normal, primary and metastatic stroma by ELISA. Metastatic & primary stroma: (^#^ *P* < 0.03)

To investigate whether CAF aggregates generated from primary or metastatic breast tumor tissues display different gene expression patterns, RNA samples were extracted from each independent aggregate culture and analyzed via RNA-Seq. The raw FASTQ files obtained from RNA-seq were analyzed via CLC Genomic Workbench to compare gene expression levels between groups. Differences in relative gene expression levels between each CAF aggregate group (normal, primary tumor, and brain metastasis) are shown as a heat map (Fig. 3a) generated through hierarchical clustering. Additionally, a list of the top differentially expressed growth factors and cytokines is shown in Supplemental Table 1. Based on gene transcript expression differences, among the consistently over expressed transcripts in the metastatic aggregates were CXCL16, CXCL12, and platelet-derived growth factor alpha. Amongst these, CXCL16 showed the highest fold change in CAFs from metastatic tumors compared to normal breast or primary tumors (5.34 and 6.436, respectively).

While reports have shown that tumors produce high levels of chemokines including CXCL12, these studies did not identify CAFs as the source within the TME.^17, 18^ High level secretion of CXCL16 from patient-derived brain metastasis CAFs has not been reported. To further validate differentially expressed transcripts from each group of aggregate, quantitative RT-PCR analyses (Fig. 3b) and IHC were performed on patient tissues and CAF aggregates (Fig. 3c). Relative changes in chemokine genes expression and proteins levels were directly related to the RNA-seq and qRT-PCR data illustrated in Fig. 3a, b. While CXCL16 can exist as either secreted or trans-membrane bound forms, only the soluble form is known to function as a chemotactic ligand for CXCR6-expressing cancer and immune cells.^19–21^ Production of the secreted form of CXCL16 was analyzed via ELISA from each representative CAF population (Fig. 3d). High levels of secreted chemokines observed in the ELISA assay from brain metastatic CAF aggregates provides a mechanism by which breast cancer cells are attracted to the metastatic brain microenvironment.

### Effects of CAFs in migration of cancer cells

**Fig. 4:**
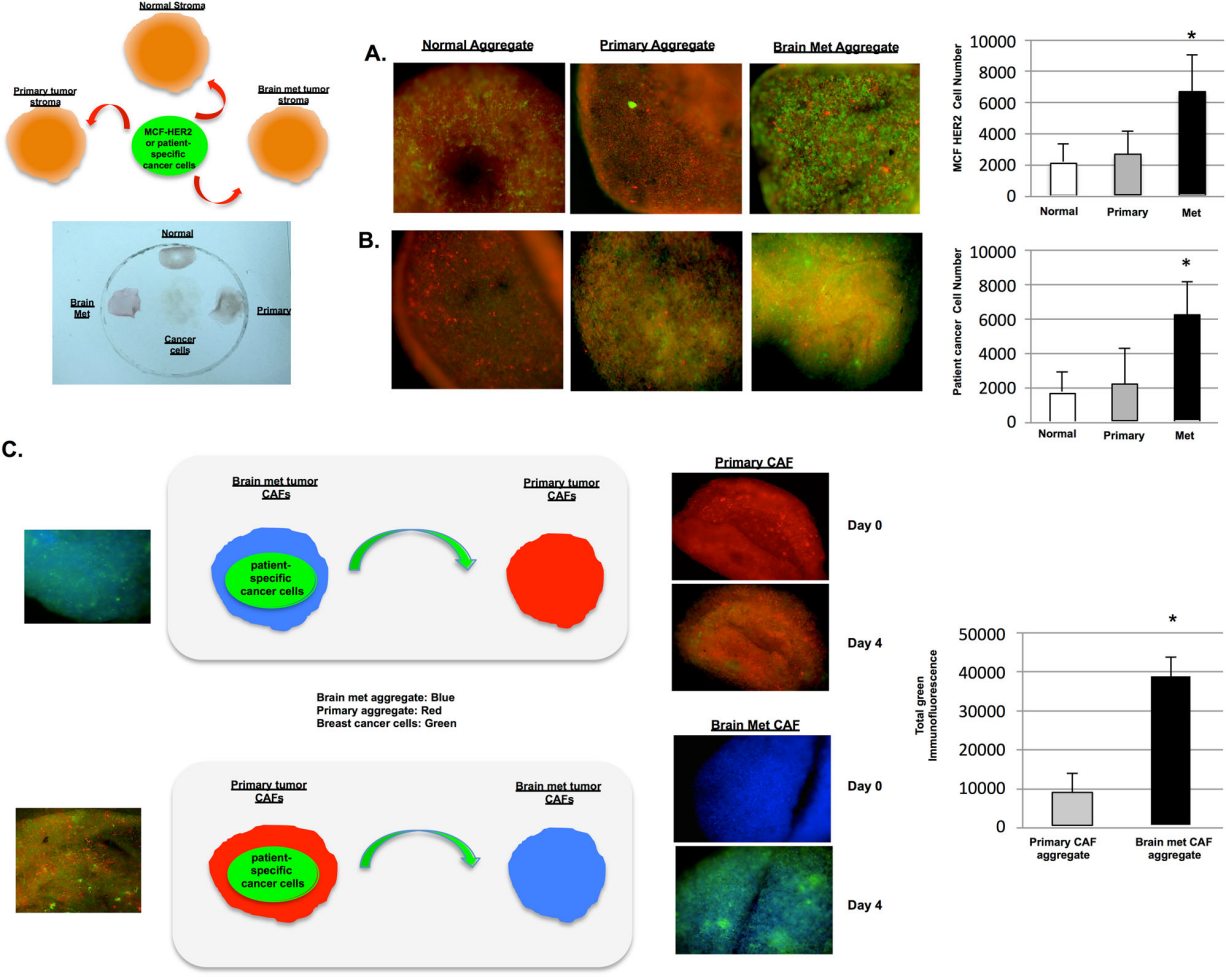
Effects of CAF in migration of cancer cells in vitro. **a** Immuno-fluorescent images comparing migration of cancer cell line MCF-Her2 (*Red*) to either normal, primary or metastatic patient-derived CAF aggregate. *Bar graph* quantifies relative migration of Her-2+ (CD340+) cancer cells to normal (BC69, BC82, BC97, and BC102), primary (BC80, BC95, BC105, and BC108), or metastatic (BC25, BC55, BC66, and BC70) CAF aggregates (*see* Table 1). **b** Immunofluorescent images comparing migration of patient-derived cancer cells to either normal, primary or metastatic patient-derived CAF aggregate. *Bar graph* demonstrates relative migratory count of cancer cells to normal (BC69, BC82, BC97, and BC102), primary (BC80, BC95, BC105, and BC108), or metastatic (BC25, BC55, BC66, and BC70) CAF aggregates. **c** Immunofluorescence images compare migration of patient-specific cancer cells (*green*) towards either Primary CAF (*Red*) or Brain Metastasis CAF (*Blue*) on Day 0 and Day 4. *Bar graph* quantifies relative total cell fluorescence of patient-specific cancer cells that have migrated to primary or brain metastatic CAF aggregate. (^#^ *P* < 0.01)

Based on our studies indicating that brain metastatic CAF aggregates produced higher levels of chemokines CXCL12 and CXCL16 as compared to normal breast fibroblasts or primary tumor CAF aggregates, we performed cancer cell migration assays (using MCF-HER2 cells or patient-derived cancer cells) to investigate the relative propensity of breast cancer cells to migrate to these different microenvironments. Each aggregate was embedded in hydrogel solution to maintain its overall 3D structure. Figure 4a shows a schematic representation for cancer cell migration in vitro and an example photograph of CAF aggregates in hydrogel with a MCF-HER2 cell line or patient-derived cancer cells embedded in the center. Based on live cell imaging, immunofluorescent microscopy, and FACS analysis, we found that significantly higher numbers of MCF-HER2 cells or patient-derived cancer cells migrated to brain metastatic CAF aggregates than primary CAF or normal breast fibroblasts aggregates (Fig. 4b and Supplemental Fig. 6).

To further explore the chemotactic activity of primary or brain metastatic CAF aggregates, we generated PKH-labeled primary (*red color*) or brain metastatic (*blue*) CAF aggregates mixed with *green color*-labeled patient-derived breast cancer cells (1:1 ratio), and positioned these aggregates against a separate CAF aggregate without cancer cells (Fig. 4c). This hydrogel system maintains the architecture of 3-D aggregates, and also allows cancer cell migration and invasion to distant locations. We consistently observed that cancer cells already associated with primary tumor CAFs still migrated out towards brain metastasis CAF aggregates. This migration took a longer period of time than the earlier results observed in Fig. 4a, b, suggesting that cancer cells were still being partially attracted by primary tumor CAFs as they migrated away towards brain metastasis CAFs. These data confirm that brain metastasis CAFs promote migration of breast cancer cells more effectively than normal fibroblasts or primary tumor CAFs.

### CXCR4 antagonist and CXCL16 neutralizing antibody treatments reduce cancer cell recruitment

**Fig. 5:**
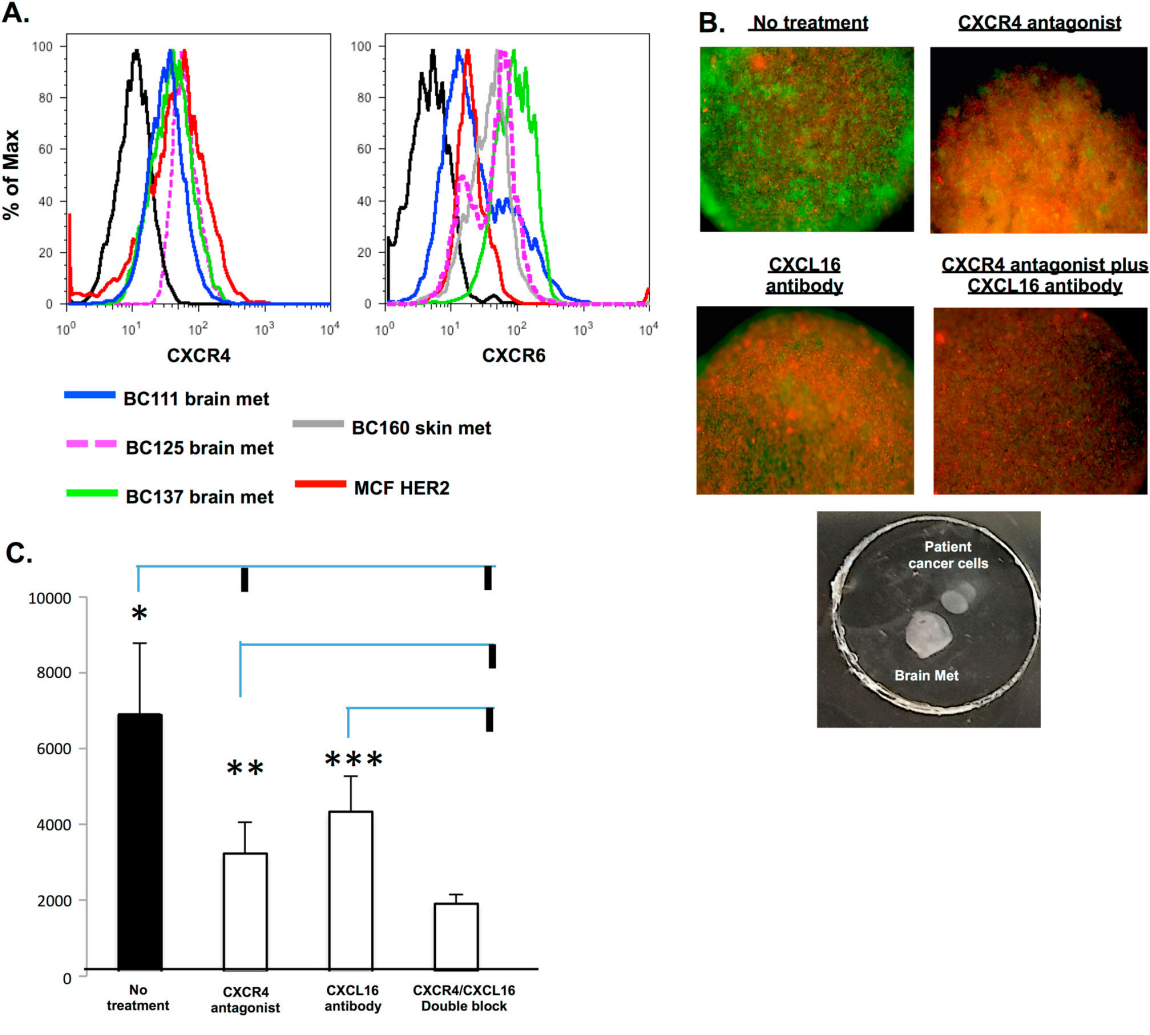
Combination of CXCR4 antagonist and CXCL16 neutralizing antibody treatment reduces cancer cell recruitment. **a** FACS analysis of relative CXCR4 or CXCR6 expression on cancer cell line MCF HER2 and patient-specific cancer cells derived from brain metastasis or skin metastasis tissue. **b** Immuno-fluorescent images demonstrate relative migration of patient-specific cancer cells (*green*) to brain metastasis stroma (*red*) with or without CXCR4 antagonist and CXCL16 neutralization in Hydrogel-Migration assay. **c**: *Bar graph* quantifies the relative migration of patient specific cancer cells that have been untreated or treated with CXCR4 antagonist, CXCL16 neutralization antibody, or a combination of both agents. An *asterisk* indicates significant differences between groups of animals (*P ≤ 0.02, **P ≤ 0.045, ***P ≤ 0.02). Each value represents the mean of 3–5 independent experiments. For the migration assay, BC25, BC55, BC66, BC70 brain metastatic CAF aggregates were independently utilized (*see* Table 1). Consistent results were obtained in repeated experiments throughout. The *vertical bold lines* branching off from the *top* of the *horizontal lines* represent statistically significant *P* values when the untreated, CXCR4 antagonist, and CXCL16 neutralizing antibody groups were individually compared with all other groups

To further investigate the importance of chemokines CXCL12 and CXCL16 secreted by metastatic CAFs on breast cancer cell migration, we analyzed the expression of cognate chemokine receptors CXCR4 and CXCR6 on patient-derived cancer cells. FACS analysis showed that patient-derived breast cancer cells expressed both CXCR4 and CXCR6 (Fig. 5a). Utilizing our hydrogel assay system, patient-derived breast cancer cells were treated with a receptor-blocking antagonist directed against CXCR4 alone or in combination with neutralizing antibody directed against CXCL16 and tested for cancer cell migration to brain metastatic CAF aggregates. Unlike CXCR4 in which a small molecule antagonist Plerixafor (Selleckchem) is available, only anti-CXCL16 neutralizing antibody is available for blocking the CXCR6–CXCL16 interaction.^22^ Indeed, CXCR4 antagonist treatment significantly reduced the ability of cancer cells to migrate to brain metastatic CAF aggregates, while CXCL16 antibody treatment was less effective than CXCR4 antagonist treatment alone (Fig. 5b, c). Combination of both inhibitors resulted in blocking cancer cell migration most significantly. These data suggest that secretion of CXCL12 and CXCL16 by CAFs plays a critical role in attracting breast cancer cells to the brain metastatic microenvironment.

## Discussion

Metastasis involves the abnormal capacity of cancer cells to migrate, and aberrant cancer cell mobility is emerging as an important area of investigation.^23^ Why breast cancer cells migrate to the brain to cause metastasis remains incompletely understood, and requires further investigation. In this study, we isolated patient-derived CAFs and cancer cells from primary human breast tumors and brain metastases, and developed 3D models to study their interactions. Based on gene and immunohistological analyses, we found that expression levels of chemokines CXCL16 and CXCL12 are significantly higher in CAFs from brain metastasis than those from primary tumors or normal breast tissues. We further demonstrated that brain metastasis CAF 3D aggregates attract patient-derived breast cancer cells much more effectively than primary tumor CAF or normal fibroblast aggregates in a hydrogel assay system. Blocking CXCR4 and/or CXCL16 neutralization, alone or in combination, resulted in significant inhibition of patient-derived cancer cells migration to brain metastatic CAF aggregates, further supporting the importance of CXCL16 and CXCL12 in migration of CTCs into brain-associated microenvironment.

CAFs have been shown to promote the growth of cancer cells via secreting various growth factors (EGF, Hepatocyte Growth Factor (HGF), FGF, Tranforming Growth Factor-alpha (TGF-*α*), Vascular Endothelial Growth Factor (VEGF) etc), and to induce invasion and metastasis of cancer cells by production of CXCL12, MMP, and transforming growth factor beta 1 (TGF-β1).^14, 24–27^ CXCL12 has been shown to play an important role in the attraction of CTCs towards future sites of metastasis,^9, 17, 27–29^ and CXCR4 (receptor for CXCL12) antagonism has been shown to inhibit tumor growth and decrease metastatic burden.^30, 31^ CXCL16/CXCR6 interaction has also been shown to promote migration and invasiveness of breast cancer cell lines, and recent studies published by Xiao *et al*. have shown that this axis can induce invasiveness based on the activation of the ERK1/2 and F-actin pathways.^20, 32, 33^ While these previous studies explored the role of these chemokine axes within cell lines and the primary TME, none of these prior investigations examined or identified the source of the chemokines within the metastatic TME. Our findings confirm the clinical significance of CXCL12 and CXCL16 in the formation of brain metastatic niche, and point to CAFs as the primary source for these chemokines.

In summary, this is the first report demonstrating the expression of both CXCL16 and CXCL12 in CAFs derived from human breast cancer metastasis in the brain. Furthermore, neutralizing antibody directed against CXCL16, alone or in combination with CXCR4 antagonist, significantly inhibited the migration of patient-derived breast cancer cells in our 3D CAF aggregate system. The unique expression of CXCL16 by brain metastasis CAFs provides an important area of cancer research that will further our understanding of metastatic progression. Our results demonstrate the importance of understanding the specific role of CAFs on metastatic progression, and possible strategies to target chemokine interactions to prevent the migration of circulating breast cancer cells to the brain.

## Materials and methods

### Culturing patient-derived cancer cells and fibroblasts

Normal breast, primary, and brain metastatic tumor tissues were obtained from patients treated at City of Hope according to guidelines approved by the City of Hope Institutional Review Board. Tissues were mechanically dissociated into small fragments (≤1 mm diameter), and cultured on 6-well tissue culture plates (Fischer Scientific) with Dulbecco’s modified Eagle’s medium (DMEM) media (D10) supplemented with 10% fetal bovine serum. For the generation of human breast CAFs, breast tumor fragments were cultured in DMEM supplemented with 10% FBS (D10 medium), penicillin/streptomycin (Invitrogen), and L‐glutamine (Invitrogen). To promote adherence of tumor fragments, tissue culture plates were coated with 0.1% gelatin (Invitrogen). CAF cultures can be passaged for up to 4 weeks, and can be frozen for later use while maintaining their mesenchyme-derived surface markers consistenly. However, to maintain similar CAF characteristics in culture, isolated CAFs were passaged no more than four times for these experiments. Breast cancer cells were cultured in calcium-free DMEM supplemented with 1% FBS, cholera toxin (10 ng/mL), bovine insulin (60 ug/mL), hydrocortisone (0.5 ug/mL), epidermal growth factor (20 ng/mL), penicillin/stremptomycin, and L-Glutamine.

### Generation of patient-derived 3D aggregate

To generate normal, primary and metastatic patient-derived 3D aggregates, cancer or stromal cells were centrifuged at 1300 RPM for 5 min into pellets in 1.5 mL microcentrifuge tubes. After removal of the supernatant, aggregates were retrieved using micropipette tips (approximately 10 ul), and expelled onto nucleopore filter membrane (0.8 µm pore size; Millipore, Billerica, MA, USA) floating on gel foam inserts (Pharmacia & Upjohn, Bridgewater, NJ, USA) supplemented with D10 medium. Following 2–4 weeks, patient-derived aggregates were harvested in culture, and utilized for downstream experimentation, and analysis in both the hydrogel migration assay and immunohistochemistry.

### Chromogenic immunohistochemistry

The composition of the extracellular matrix and collagen architecture was examined by chromogenic immunohistochemistry. Three-micrometer paraffin embedded sections of tissues or aggregates were deparaffinized in xylene, and subsequently rehydrated in descending concentrations of ethanol. Antigen retrieval was performed utilizing a 1× DIVA citrate buffer (pH = 6.0) in a Biocare Decloaker (Biocare Medical, Concord, CA, USA) for 30 s at 125 °C, then 3.5 min at 90 °C. The following primary antibodies were incubated on the tissue or aggregate sections for 1 h: *α*-smooth muscle actin (mouse monoclonal clone 1A4) and vimentin (mouse monoclonal clone V9), both from Dako, Carpinteria, CA, USA; Collagen IV (Mouse monoclonal Col94), Biocare Medical, Walnut Creek, CA, USA, Fibronectin (mouse monoclonal sc-271098), Santa Cruz biotechnology, Santa Cruz, CA, USA); and FAP (Rabbit polyclonal clone AB2), Sigma-Aldrich, St Louis, MO, USA. Based upon the species of each primary antibody, the following secondary antibodies were incubated on the tissue or aggregate of interest for 20 min: MACH 2 Mouse HRP-polymer (Biocare Medical, Concord, CA, USA) or MACH 2 Rabbit HRP-polymer. Following incubation and a rinse in Tris-Buffered Saline solution buffer, the DAB Chromogen Kit from Biocare Medical was used to illustrate the marker of interest.

### Fluorescent immunohistochemistry

Patient-derived aggregates and tissue sections were paraffin embedded, and de-paraffinized in Xylene. Anti-Cytokeratin (mouse monoconal), and Vimentin (mouse monoclonal clone V9) antibodies were purchased from Dako (Carpinteria, CA, USA), anti-Ki67 (rabbit monoclonal) antibody was purchased from Biocare (Concord, CA, USA). The fluorophores Cy3, FITC, and Alexa 647 conjugates were used at a concentration of 1 μg/mL (Biocare). Imaging was performed through the Zeiss Axio Observer. The chemokine composition of the patient-derived aggregate was identified by using primary antibodies on the tissue or aggregate of interest for 1 h: Vimentin (mouse monoclonal clone V9), SDF-1 (rabbit monoclonal; Santa Cruz Biotechnology, Santa Cruz, CA, USA) and the CXCL16 (rabbit monoclonal; abcam, Cambridge, MA, USA).

### Histological and immunofluorescent microscopy

Imaging of the 3 μm formalin-fixed paraffin embedded tissues and aggregates were performed using an Olympus BX51WI fixed-stage upright microscope equipped with the Vectra platform (Perkin-Elmer, Waltham, MA, USA). Immunofluorescent imaging of the hydrogel assay was performed utilizing a Zeiss Axiovert 200 M-wide field fluorescence microscope equipped with the RTE CCD 1300-XHS camera.

### Fluorescent cell membrane labeling

To elucidate and differentiate both the cancer and stromal cells migrating within the hydrogel, PKH26 Red Fluorescent membrane stain (Sigma Aldrich) was used to stain stromal cells, and PKH67 Green Fluorescent membrane stain (Sigma Aldrich) was utilized to stain cancer cells. Both stromal and cancer cells numbering 1.0–2.0 × 10^6^ cells/mL were re-suspended in 1× PBS. One-micromolar of either PKH67 green dye or PKH26 red dye was mixed with 500 μl of Diluent C to a single cell suspension in a 2 mL centrifuge tube in which 0.5 μM PKH26/67 final dye concentration was used. The cells were then incubated in the dark for 5 min at 37 °C. Subsequent washing of the cells in 800 μL of FBS quenched the staining reaction, and cell mixtures were pelleted via centrifugation at 1000 RPM for 10 min. Brain metastasis stromal cells were labeled blue with Wheat Germ Agglutinin, Alexa Fluor^®^ 350 Conjugated from Invitrogen. The cells were incubated for 10 min at 37 °C at a starting concentration of 5.0 μg/mL, then washed twice in HBSS buffer. Following two washes in HBSS buffer, cells are pelleted, and placed onto the Millipore membrane filter /Foam system that served as the vehicle for the cells to form 3D aggregates.

### Hydrogel migration assay

The HyStem hydrogel kit (ESI-Bio) was utilized to create the proper scaffold for migration assays. Hydrogel is a hyaluronan-based gel that effectively crosslinks thio-reactive poly(ethylene glycol) diacrylate, and was formulated according to manufacturer’s instructions consisting of three components: thiol-modified hyaluronan (Glycosil^®^), Thiol-reactive PEGDA crosslinker (Extralink^®^), and thiol-modified collagen (Gelin-S^®^). Glycosil and Gelin were separately dissolved in 1.0 mL of deionized water, and allowed to dissolve for 1 h. The cross-link agent Extralink^®^ was then dissolved with 0.5 mL of deionized water. Following the solvation of Glycosil and Gelin-S, the compounds were mixed in a 1:1 ratio. To form the hydrogel, Extralink was added to the Glycosil + Gelin-S mixed in a 1:4 volume ratio. The heterogeneous mixture of Gelin-S, Glycosil, and Extralink were then added in 60 uL aliquots to a 35 mm tissue culture dish. Once the gel became opaque in appearance, D10 medium was added onto the gel and surrounding area. The dish was then placed in a humidified 37 °C incubator with O_2_ and 10% CO_2_. The relative migration of PKH green-labeled cancer cells was measured to normal, primary, or metastatic patient aggregates that were labeled with PKH red. Furthermore, a heterogeneous aggregate composed of cancer cells combined with either brain metastatic stroma or primary stroma was created. The relative migration of cancer cells within the aggregate system was measured through both fluorescent microscopy and flow cytometry.

### CXCR4 antagonist and CXCL16 neutralization hydrogel migration assay

CXCR4 antagonist Plerixafor (Selleckchem) and CXCL16 neutralizing antibody were prepared in a 1× PBS (Dulbeccos’s) at a stock concentration of 500 mM. Cell lines or patient-specific cells were washed in 1× PBS and re-suspended in 500 µL of 1× PBS supplemented with 100 mM of Plerixafor and/or 0.25 µg of anti-CXCL16 (purchased from Selleckchem and R & D Systems). The cancer cell-antagonist mixture was then incubated for 2 h in a humidified incubator at 37 °C with O_2_ and 10% CO_2_. The antagonized cells were then pelleted at 1200 RPM for 5 min, and utilized for downstream hydrogel-migration assays.

### RNA-seq library preparation and data analysis

Patient-derived aggregates were mechanically dissociated into a single-cell suspension, subsequently the aggregates’ RNA was purified using the RNeasy Plus Micro Kit (Qiagen) according to the manufacturer’s protocol. The purified RNA libraries were then sequenced using the Illumina HiSeq 2000 at the City of Hope Integrative Genomics Core. The RNA-seq sequence reads were aligned with Human genome (hg19) using open source RNA-seq alignment tool Tophat (v2.0.8b). The alignment results were converted to RNA-seq gene expression measurement as RPKM (reads/kilo base of total exon length/million mapped) using CLC Genomic Workbench, and normalized to gene models in the NCBI Ref Seq database. Maximum expression <0.05 RPKM was used to filter and exclude very low expressing genes. An EDGE test was then utilized to compare the gene expression between sample groups. The EDGE method was developed specifically for two-group comparisons in situations where many features are studied simultaneously but only a few biological replicates are available for each of the experimental groups (i.e., RNA-seq). Following the EDGE test, significantly differentially expressed genes characterized as cytokines or growth factors were selected (Supplemental Table 1).

### Growth factor expression in stroma by reverse transcriptase quantitative PCR

RNA was extracted from single cell suspensions utilizing an RNEasy RNA isolation kit from Qiagen. cDNA was prepared with a cDNA Super Script III RT kit (Thermo Scientific). For semi-quantitative PCR assays, Promega Master Mix was used to amplify growth factor genes. Cycling conditions consisted of a denaturation step for 5 min at 95 °C, followed by 50 cycles of denaturation (95 °C for 1 min), annealing (72 °C for 30 s) and extension (72 °C for 1 min), with final elongation cycle of 10 min at 72 °C. Quantitative PCR was performed with BioRad CFX96 and SYBR Green PCR Master Mix (Applied Biosystems).

The following primers for amplification for RT-qPCR of growth factors were:

FGF1 forward: 5′-CACATTCAGCTGCAGCTCAG-3′

FGF1 reverse: 5′-TGCTTTCTGGCCATAGTGAGTC-3′

FGF2 forward: 5′-CTTCTTCCTGCGCATCCACC-3′

FGF2 reverse: 5′-CACATACCAACTGGTGTATTTC-3′

EGF forward: 5′-TGGATGTGCTTGATAAGCGG-3′

EGF reverse: 5′ACCATGTCCTTTCCAGTGTGT-3′

IGF-1 forward: 5′-TGGATGCTCTTCAGTTCGTG-3′

IGF-1 reverse: 5′-TGGTAGATGGGGGCTGATAC-3′

CXCL12 forward: 5′-GGGCTCCTGGGTTTTGTATT-3′

CXCL12 reverse: 5′-GTCCTGAGAGTCCTTTTGCG-3′

CXCL16 forward: 5′-GGCCCACCAGAA GCATTTAC-3′

CXCL16 reverse: 5′-CTGAAGATGCCCCCTCTGAG-3′

### Human CXCL16 ELISA

Patient-derived stromal cells were cultured on 6-well tissue culture plates. 1 mL of media was collected from each well, and stored at −20 °C. Supernatant media of patient-derived stromal cells were collected, and analyzed for human CXCL16 via ELISA (Peprotech). ELISA plates were prepared per Peprotech protocol. Assays were performed in triplicate, and absorbance at 405 nm was read on a Wako/Tecan immuno-plate reader.

## Electronic supplementary material

Supplementary Information
