# Supplementary material for: Human brain metastatic stroma attracts breast cancer cells via chemokines CXCL16 and CXCL12
Source: NPJ Breast Cancer. 2017 Mar 2;3:6. doi: 10.1038/s41523-017-0008-8 (PMC5460196; doi:10.1038/s41523-017-0008-8)
Supplement: Supplementary file 1 — Supplementary Information [file 41523_2017_8_MOESM1_ESM.pdf]

Supplementary Figure 1

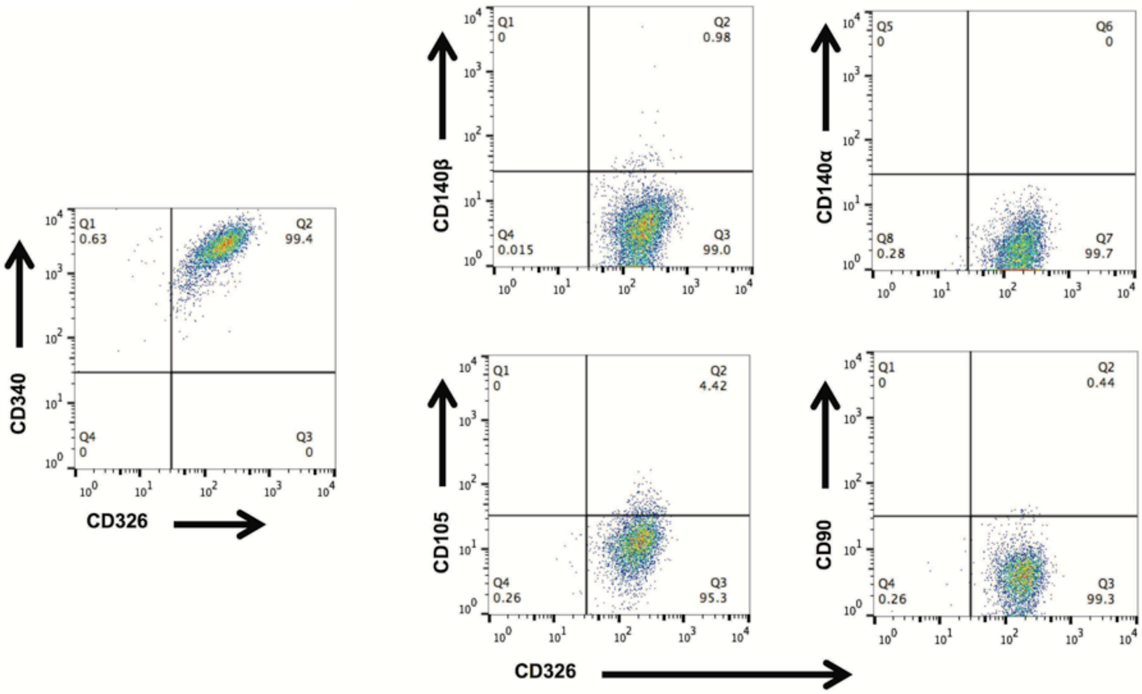

Supplemental Figure 1. FACS analysis showing cultured CD326+ CD340+ (Her2) patient-derived cancer cells. Compared to normal stroma and CAFs, CD326+ CD340+ patient-derived breast cancer cells do not express CD105, CD90, CD140β, and CD140α surface markers that are expressed by CAFs.

Supplementary Figure 2

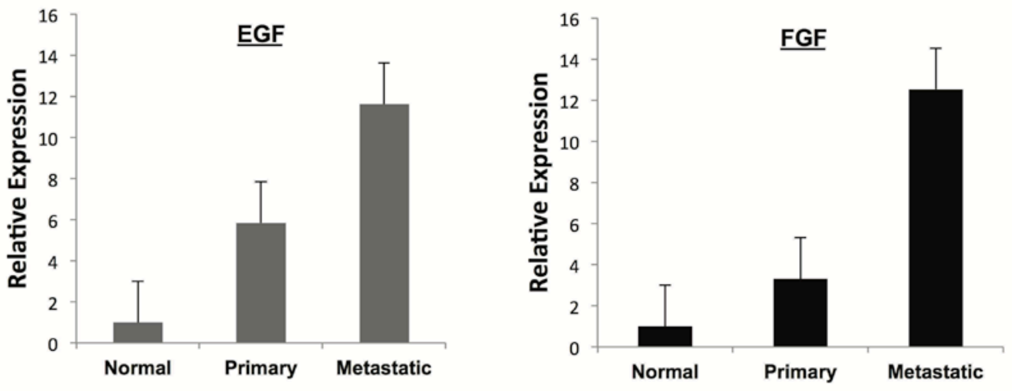

Supplemental Figure 2. Quantitative RT-PCR analyses of normal, primary and metastatic patient-derived stroma cells. Data represent mRNA expression in stroma cells derived from normal breast tissue (BC97, BC131, and BC227) primary breast tumor tissue (BC105, BC108, BC221) and brain metastatic tissue (BC25, BC55, and BC70).

Supplementary Figure 3

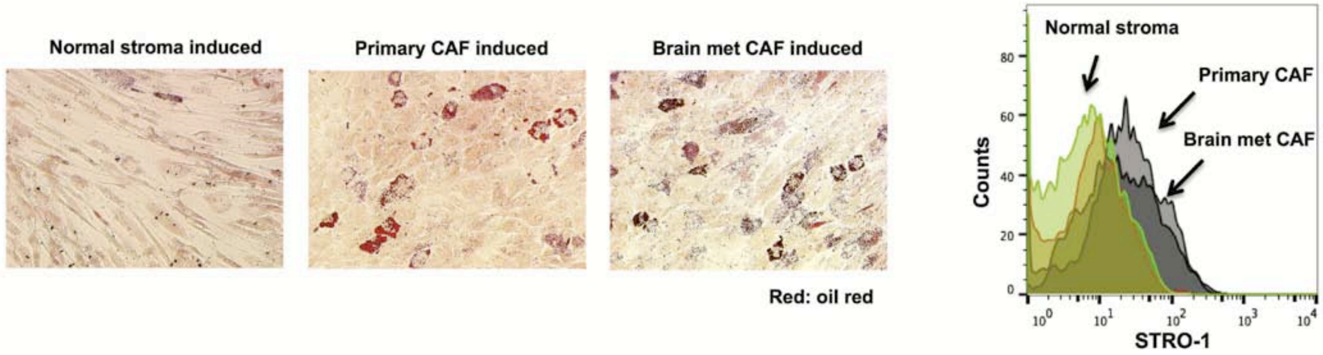

Supplemental Figure 3. Representative images of CAF tissue culture showing that both primary and brain met stroma populations contain STRO-1+ (MSC marker) expressing cells. Positive Oil Red O staining from primary and brain met CAF show their ability to undergo adipogenesis.

Supplementary Figure 4

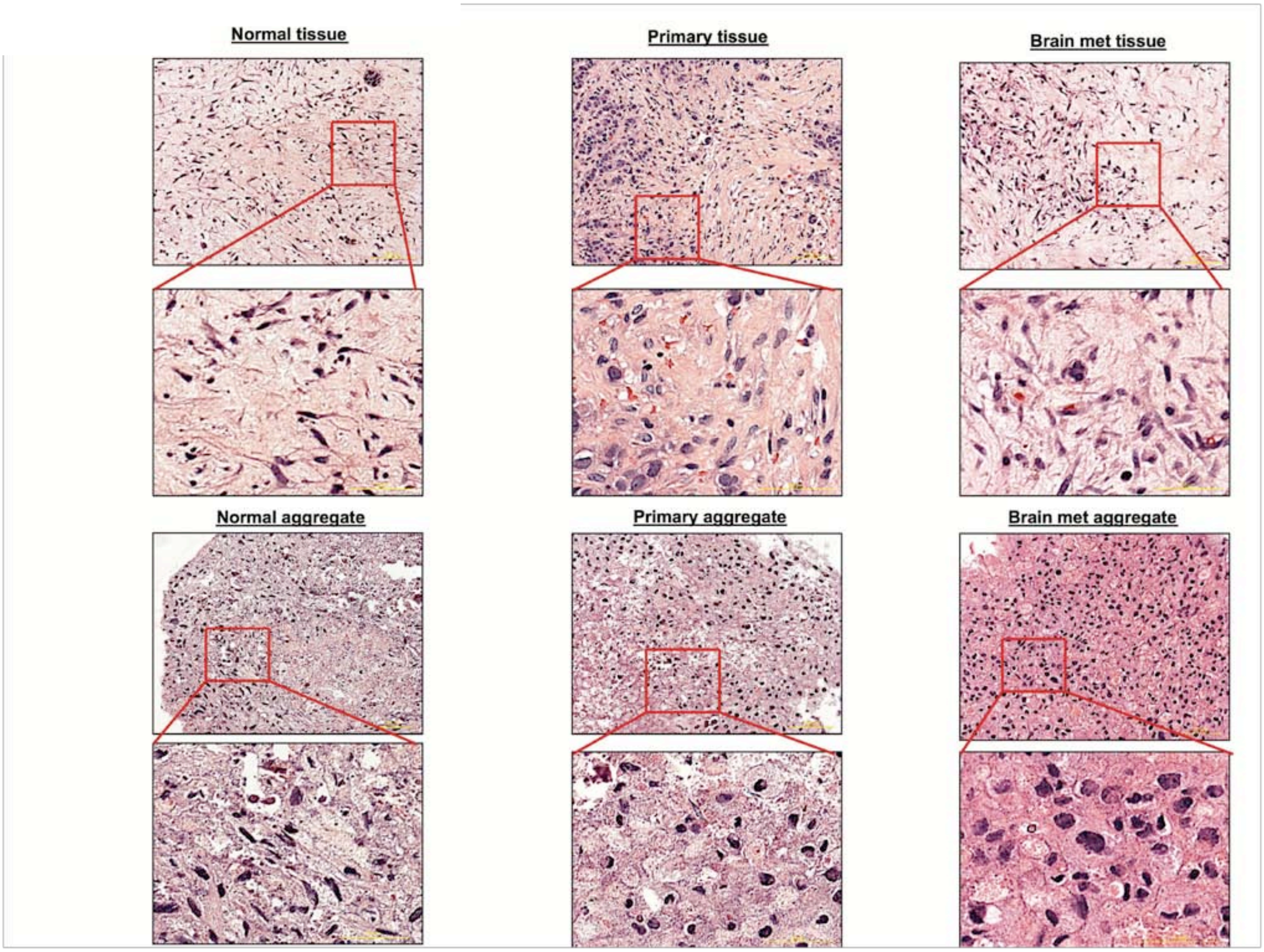

Supplemental Figure 4. Representative H&E histological images illustrate the morphological similarities between patient tissue samples and 3D patient-derived stromal aggregates. Scale bars of zoomed images represent 50µm.

Supplementary Figure 5

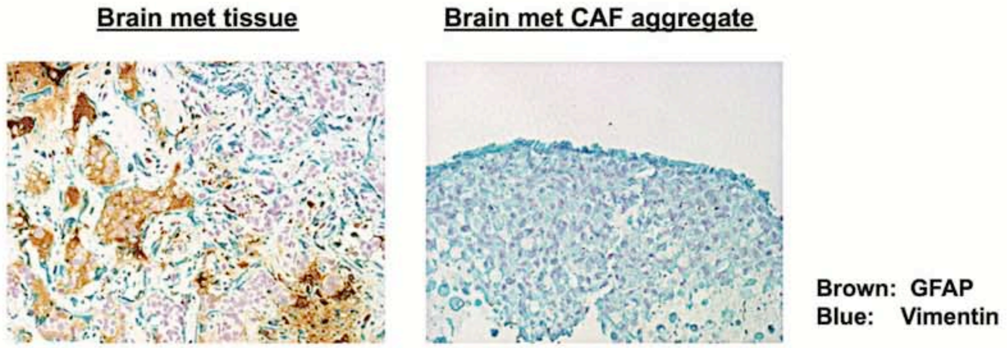

Supplemental Figure 5. Immunohistochemical antibody staining of Glial Fibrillary Acidic Protein (GFAP) in brain met patient-derived tissue and aggregate composed purely of brain-met stroma. The figure shows that the brain met CAF aggregate is GFAP negative, demonstrating that the isolated brain met stroma are not of glial or neural derivation.

Supplementary Figure 6

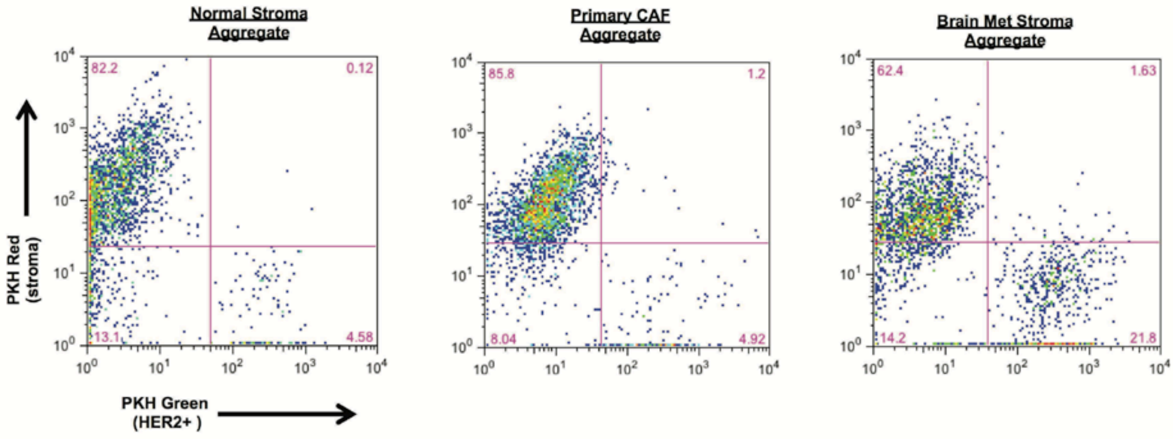

Supplemental Figure 6. FACS analysis demonstrating the relative migration of PKH green-labeled cancer cells to normal, primary or metastatic patient aggregates that were labeled with PKH red. 24 hours post-incubation, higher percentage of patient-specific cancer cells migrated to brain metastatic CAF aggregates than primary CAF or normal breast fibroblasts aggregates.

Primary vs. Normal

Symbol	Entrez Gene Name	Exp p-value	Exp Fold Change
OGN	osteoglycin	0.000	20.174
FGF10	fibroblast growth factor 10	0.002	18.125
TGFB2	transforming growth factor beta 2	0.000	8.068
CXCL10	C-X-C motif chemokine ligand 10	0.013	7.12
HGF	hepatocyte growth factor	0.003	2.838
MDK	midkine (neurite growth-promoting factor 2)	0.013	2.151
KITLG	KIT ligand	0.059	1.634
VEGFC	vascular endothelial growth factor C	0.042	-1.963
SPP1	secreted phosphoprotein 1	0.015	-2.348
NGF	nerve growth factor	0.038	-2.544
INHBA	inhibin beta A	0.023	-3.036
LIF	leukemia inhibitory factor	0.003	-3.077
CXCL8	C-X-C motif chemokine ligand 8	0.056	-3.549
DKK1	dickkopf WNT signaling pathway inhibitor 1	0.008	-4.189
IL11	interleukin 11	0.051	-4.379
CTGF	connective tissue growth factor	0.002	-4.49
IL6	interleukin 6	0.001	-8.292
CXCL2	C-X-C motif chemokine ligand 2	0.010	-10.812
CSF3	colony stimulating factor 3	0.011	-15.91

Brain Met vs. Normal

Symbol	Entrez Gene Name	Exp p-value	Exp Fold Change
CX3CL1	C-X3-C motif chemokine ligand 1	0.000	256.042
AGT	angiotensinogen	0.000	247.041
PGF	placental growth factor	0.000	12.237
PDGFA	platelet derived growth factor subunit A	0.000	8.859
TGFB2	transforming growth factor beta 2	0.002	5.39
CXCL16	C-X-C motif chemokine ligand 16	0.001	5.341
PTN	pleiotrophin	0.000	5.036
SCG2	secretogranin II	0.048	4.85
NDP	Norrie disease (pseudoglioma)	0.031	4.664
FGF1	fibroblast growth factor 1	0.125	3.496
ANGPT1	angiopoietin 1	0.059	2.795
CXCL12	C-X-C motif chemokine ligand 12	0.069	1.814
GRN	granulin	0.041	1.355
CLEC11A	C-type lectin domain family 11 member A	0.025	-1.654
GAS6	growth arrest specific 6	0.017	-2.221
NGF	nerve growth factor	0.060	-2.231
VEGFC	vascular endothelial growth factor C	0.013	-2.273
INHBA	inhibin beta A	0.083	-2.294
IL33	interleukin 33	0.071	-2.367
SPP1	secreted phosphoprotein 1	0.005	-2.735
DKK1	dickkopf WNT signaling pathway inhibitor 1	0.041	-2.809
IL6	interleukin 6	0.075	-2.901
FGF7	fibroblast growth factor 7	0.055	-3.568

Brain Met vs. Primary

Symbol	Entrez Gene Name	Exp p-value	Exp Fold Change
CX3CL1	C-X3-C motif chemokine ligand 1	0.000	323.562
AGT	angiotensinogen	0.000	120.006
PGF	placental growth factor	0.000	12.799
PDGFA	platelet derived growth factor subunit A	0.000	10.296
FGF1	fibroblast growth factor 1	0.031	7.398
SCG2	secretogranin II	0.016	7.100
EREG	epiregulin	0.063	6.756
CXCL16	C-X-C motif chemokine ligand 16	0.001	6.436
CSF3	colony stimulating factor 3	0.098	6.097
CTGF	connective tissue growth factor	0.000	5.356
PTN	pleiotrophin	0.000	3.671
ANGPT1	angiopoietin 1	0.027	3.549
IL6	interleukin 6	0.139	2.858
LIF	leukemia inhibitory factor	0.063	2.052
TIMP1	TIMP metallopeptidase inhibitor 1	0.026	-1.509
CLEC11A	C-type lectin domain family 11 member A	0.025	-1.656
IL32	interleukin 32	0.249	-1.662
KITLG	KIT ligand	0.009	-1.992
HGF	hepatocyte growth factor	0.024	-2.179
TYMP	thymidine phosphorylase	0.020	-2.217
NAMPT	nicotinamide phosphoribosyltransferase	0.038	-2.337
CXCL6	C-X-C motif chemokine ligand 6	0.180	-2.552
MDK	midkine (neurite growth-promoting factor 2)	0.001	-2.837
CXCL10	C-X-C motif chemokine ligand 10	0.098	-3.106
FGF10	fibroblast growth factor 10	0.097	-3.253
BMP2	bone morphogenetic protein 2	0.024	-3.523
IL1RN	interleukin 1 receptor antagonist	0.081	-3.586
CCL7	C-C motif chemokine ligand 7	0.053	-3.651
FGF7	fibroblast growth factor 7	0.025	-4.264
OGN	osteoglycin	0.002	-9.248

Supplemental Table 1. A list of cytokines and growth factors that are differentially expressed between normal stroma, primary tumor and brain met CAF aggregates. Genes are ranked based on fold change.
